# Dental Economics

**Published:** 1885-07

**Authors:** J. H. Paine

**Affiliations:** Middletown, O.


### Dental Economics.

BY DR. J. H. PAINE, MIDDLETOWN, O.

Read before the Mad River Valley Dental Society, Dayton, O., May 19th, 1885.

In the dental profession we may appropriately, I think, lay down the basis of our useful science, and name it dental economy; and as there are several and diverse phases we will include all under the head: Dental Economics.

First, we claim for dentistry a conservative element. To destroy is easy,—requires no learning, no art nor science; but to save requires scientific knowledge, art in manipulation, and faith in evolution,—that kind of evolution which, working jointly with the forces of nature, evokes a better man, physically, intellectually, and morally. To illustrate: It is one of the economics of operative dentistry to reconstruct a diseased and carious set of natural teeth. Many of them are impaired by decay. One, two, three, and even five cavities are not unfrequently met with in an individual tooth. Whoever transforms the oral cavity from an unclean, discolored, unhealthy, disgraceful pool of noxious disease-breeding, health-ruining, uninviting vortex, wherein revel bacteria and animalculæ, into a restored set of teeth, well plugged, cleaned of carbonate of lime, and rescued from gnawing, rankling bacteria and animalculæ, is an economist, *par excellence* to his race, and stands in the forefront among evolutionists, whether of the body, mind, or soul. We believe that well-filled, clean teeth indicate intelligence. In every community he, she, or they are the best looking, most tasteful, and most godly who take the best care of their teeth. Hence, we prove our first proposition that the capable dentist practices the noblest phase of dental economics, when he gives most attention to saving the natural teeth. Useful as artifical teeth are, they should always stand aside, or in the process of manufacture, should be at once laid on the laboratory table, when there is a waiting cavity in a natural tooth in the operating room. One of our best dentists never neglects a natural tooth that is known to be waiting for his art preservative for any other work, certainly not for plate work. The latter will do well enough when there is nothing better, nothing higher, nothing more ennobling. True dental economics has written all over its diploma, save! save! and, in parenthesis—only quacks needlessly destroy. But some man will say, there are so many filthy, non-appreciating, non-paying patrons, in every community, that the dentist can not afford to bestow the care, skill, and persistency necessary to evolutionize this dental apparatus from filthiness, *per se*, to sweetness, *de facto*. Yet we may teach all nations, baptizing them with truthful teaching. Instruction is golden. Therein lies all the difference that pervades the immense distances between savage and cultured life, between a slum in Madagascar, and a professor in Harvard University.

We wish to rank next to intelligence on the part of the dentist, in his own person and individuality, exact and scrupulous cleanliness. What if he be poor? water is free; tooth brushes are cheap; *creta preparata* is not costly; perfection soap is attainable; shoe blacking pays and tells that he is not only a dentist, but a gentleman as well. If he practice the preaching he orates to his patients, the preaching will do more good, and in fact will do only the good that is represented in practice.

We most solemnly adjure the younger brethren of the profession to abstain from every untidy, coarse form of amusement. Playing base ball, grooming horses, etc., are not elevating in ethics, nor helpful in dentistry. A brisk walk for exercise, a good book for company; and a patient in the operating chair should give the dentist more pleasure than anything else beside. It were well to have it lettered in gold on the wall, where it will constantly inspire you, destroy not, save if possible, SAVE! But some man will say, how shall I save? We answer: save by removing the diseased part and the dead. There are many cases the dentist sees not until death has begun its work. Toothache is usually death of the pulp begun. Now it is not always necessary to remove the entire pulp to rescue from death the entire tooth. It is not always necessary for the general surgeon to remove the entire limb to save the body. Hence, as to topical applications, we reject arsenious acid. It is no longer used by the far-seeing dental surgeon. Be as cautious as he will, it will get where he does not want it to go, and even years afterward, if not earlier, will exhibit its "killing" work, in the translucent appearance of the dentine, the blue-edged color line about the cervical wall, and the worse than all beside, condition of encystment of the periosteum in the alveolar process, and the inevitable break-down of its ossific structure; and irremediable extraction must follow ere peace comes to that house.

What the dentist wants in his practice in saving the natural teeth is not death, but life. Save the pulp if possible. If it dies, remove as much of it as you can, using great care to not thrust the instrument through the foramen and inoculate the alveolar process with the pus of the dead pulp, so that alveolar abscess be superinduced; and not only would we then have dead pulp of the tooth, dead periostem, or parts about the apex, but presently dead tooth itself, and then mechanical dentistry is invoked, and Ichabod should be written on our wall; for we have filled the tooth and it is lost.

